# Supplementary material for: In Vitro and In Silico Pharmacological and Cosmeceutical Potential of Ten Essential Oils from Aromatic Medicinal Plants from the Mascarene Islands
Source: Molecules. 2022 Dec 8;27(24):8705. doi: 10.3390/molecules27248705 (PMC9788324; doi:10.3390/molecules27248705)
Supplement: Supplementary file 1 [file molecules-27-08705-s001.zip › molecules-2046897-supplementary (2).pdf]

In Vitro and In Silico Pharmacological and Cosmeceutical Potential of Ten Essential Oils from Aromatic Medicinal Plants from the Mascarene Islands

Bibi Sharmeen Jugreet [1], Namrita Lall [2,3,4], Isa Anina Lambrechts [2], Anna-Mari Reid [2], Jacqueline Maphutha [2], Marizé Nel [2], Abdallah H. Hassan [5], Asaad Khalid [6,7], Ashraf N. Abdalla [8], Bao Le Van [9,10,*] and Mohamad Fawzi Mahomoodally [1,11,12]

[1] Department of Health Sciences, Faculty of Medicine and Health Sciences, University of Mauritius, Réduit, Mauritius
[2] Department of Plant and Soil Sciences, University of Pretoria, Pretoria, South Africa
[3] School of Natural Resources, University of Missouri, Columbia, MO, United States.
[4] College of Pharmacy, JSS Academy of Higher Education and Research, Mysuru, India
[5] Chemistry Department, College of Education, Salahaddin University, Erbil, Iraq
[6] Substance Abuse and Toxicology Research Center, Jazan University, P.O. Box: 114, Jazan 45142, Saudi Arabia
[7] Medicinal and Aromatic Plants and Traditional Medicine Research Institute, National Center for Research, P. O. Box 2404, Khartoum, Sudan
[8] Department of Pharmacology and Toxicology, College of Pharmacy, Umm Al-Qura University, Makkah 21955, Saudi Arabia
[9] Institute of Research and Development, Duy Tan University, Da Nang, Vietnam
[10] Faculty of Natural Sciences, Duy Tan University, Da Nang, Vietnam
[11] Center for Transdisciplinary Research, Department of Pharmacology, Saveetha Dental College, Saveetha Institute of Medical and Technical Science, Chennai 600077, India
[12] Centre of Excellence for Pharmaceutical Sciences, North-West University, Private Bag X6001, Potchefstroom, 2520, South Africa
* Correspondence: vnble@duytan.edu.vn

Table S1. Percentage yields and chemical composition of major components of the studied essential oils (EOs).

EOs	% Yield (w/w)[a]	Major EO components[b]
CAF	0.24	Limonene (84.3%), 9-octadecanoic acid (3.9%), germacrene D (2.5%), myrcene (2.3%)
CAL	0.17	Sabinene (38.1%), citronellal (13.7%), *(E)*-β-ocimene (11.6%), citronellyl acetate (5.2%), terpinen-4-ol (5.1%), γ-terpinene (4.0%), β-pinene (3.0%), myrcene (3.2%), limonene (2.6%), α-terpinene (2.3%), α-pinene (2.0%)
CC	0.73	1,8-cineole (54.0%), sabinene (14.6%), α-terpineol (9.8%), α-pinene (4.8%), terpinen-4-ol (3.4%), β-pinene (3.5%)
CL	1.21	Turmerone (31.4%), ar-turmerone (16.1%), turmerol (14.6%), terpinolene (11.0%), α-zingiberene (5.2%), β-sesquiphellandrene (4.8%), β-caryophyllene (3.5%)
MC	0.35	Octanoic acid (78.9%), hexanoic acid (11.3%), [octanoic acid, methyl ester] (5.4%)
PA	0.23	Carvacrol (17.9%), δ−3-carene (15.2%), camphor (12.9%), *p*-cymene (9.9%), γ-terpinene (6.6%), β-caryophyllene (6.1%), β-selinene (4.2%), α-terpinene (4.1%), *trans*-β-bergamotene (4.0%)
PC	0.09	Myristicin (40.3%),1,3,8-*p*-dimenthatriene (17.9%), β–phellandrene (15.0%), myrcene (4.2%), α, *p*-dimethylstyrene (3.7%), terpinolene (2.6%), limonene (2.5%)
PS	0.77	Myrcene (62.2%), germacrene D (7.8%), limonene (3.4%), 9-octadecanoic acid (3.1%), β–phellandrene (2.9%), δ-cadinene (2.9%)
SC	0.03	*(E)*-β-ocimene (24.4%), *(Z)*-β-ocimene (10.7%), α-guaiene (12.6%), β-selinene (9.7%), myrcene (7.8%), δ-guaiene (7.2%), selin-11-en-4 α-ol (3.8%), α-selinene (3.1%)
SS	0.04	β-pinene (21.3%), α-pinene (8.9%), γ-terpinene (7.9%), limonene (7.7%), *p*-cymene (5.9%), β-selinene (3.8%), selin-11-en-4 α-ol (3.6%), β-caryophyllene (3.5%), α-selinene (3.4%), δ-cadinene (2.9%), 1-epi-cubenol (2.2%), terpinolene (2.1%), α-terpineol (2.1%)

CAL: *Citrus aurantium* leaf, CAF: *Citrus aurantium* fruit (peel), CC: *Cinnamomum camphora*; CL: *Curcuma longa*, MC: *Morinda citrifolia*, PA: *Plectranthus amboinicus*, PC: *Petroselinum crispum*; PS: *Pittosporum senacia*; SC: *Syzygium coriaceum*; SS: *Syzygium samarangense*; [a]w/w per 100 g of plant materials; [b]identified by GC-MS/GC-FID [1,2].

			% Abundance									
**No.**	**Compounds**	**RRI[a]**	**CAF**	**CAL**	**CC**	**CL**	**MC**	**PA**	**PC**	**PS**	**SC**	**SS**
1	Tricyclene	1009	-[b]	-	-	-	-	-	-	tr[c]	-	-
2	α-Pinene	1023	0.6	2.0	4.8	0.1	-	0.7	1.3	0.4	-	8.9
3	α-Thujene	1026	-	0.7	0.9	-	-	0.6	-	0.1	-	0.4
4	α-Fenchene	1057	-	-	-	-	-	tr	-	-	-	0.3
5	Camphene	1068	-	-	0.3	-	-	0.8	tr	tr	-	1.1
6	β-Pinene	1111	tr	3.0	3.5	-	-	0.1	0.8	0.9	-	21.3
7	Sabinene	1124	0.4	38.1	14.6	-	-	0.1	0.2	1.3	-	-
8	δ-3-Carene	1157	-	-	-	0.2	-	15.2	tr	0.1	-	-
9	Myrcene	1165	2.3	3.2	1.3	0.2	-	1.2	4.2	62.2	7.8	0.7
10	α-Phellandrene	1168	-	0.1	-	0.5	-	0.8	1.2	-	-	0.2
11	Pseudolimonene	1173	-	tr	-	-	-	-	0.1	-	-	-
12	α-Terpinene	1183	0.1	2.3	0.8	0.6	-	4.1	-	-	-	0.4
13	2-Heptanone	1184	-	-	-	-	0.1	-	-	-	-	-
14	Hexadecanoic acid, methyl ester	1188	-	-	-	-	1.4	-	-	-	-	-
15	Limonene	1201	84.3	2.6	0.7	0.2	0.1	1.2	2.5	3.4	0.2	7.7
16	β -Phellandrene	1210	0.4	0.7	-	-	-	0.5	15.0	2.9	tr	0.2
17	1,8-Cineole	1211	-	-	54.0	0.7	-	-	-	-	-	-
18	*(Z)*-β-Ocimene	1237	-	0.3	tr	-	-	0.1	tr	-	10.7	0.5
19	γ-Terpinene	1249	0.1	4.0	1.3	0.1	-	6.6	0.3	tr	-	7.9
20	*(E)*-β-Ocimene	1255	0.3	11.6	0.2	-	-	0.1	0.2	0.4	24.4	0.1
21	Vinyl benzene (=Styrene)	1263	-	-	-	-	-	-	-	-	-	tr
22	*p*-Cymene	1276	-	0.7	0.3	0.3	-	9.9	0.4	0.1	tr	5.9
23	Terpinolene	1286	tr	1.0	0.3	11.0	-	1.0	2.6	0.1	0.1	2.1
24	Octanal	1291	0.1	-	-	-	-	-	-	-	-	-
25	(Z)-3-Hexenyl acetate	1319	-	-	-	-	-	-	-	-	-	tr
26	cis-Alloocimene	1376	-	-	-	-	-	-	-	-	0.5	-

27	Octanoic acid, methyl ester	1391	-	-	-	-	5.4	-	-	-	-	-
28	3-Octanol	1391	-	-	-	-	-	0.1	-	-	-	-
29	1,3,8-*p*-dimenthatriene	1403	-	-	-	tr	-	-	17.9	-	-	-
30	Rosefuran	1404	-	-	-	-	-	-	-	-	tr	-
31	α-Fenchone	1408	-	-	-	-	-	0.2	-	-	-	-
32	Perillen	1426	-	-	-	-	-	-	-	0.1	-	-
33	Octanoic acid, ethyl ester	1436	-	-	-	-	0.1	-	-	-	-	-
34	α, *p*-dimethylstyrene	1447	-	-	-	0.3	-	0.1	3.7	-	-	tr
35	1-Octen-3-ol	1450	-	-	-	-	-	0.6	-	-	-	-
36	α-Cubebene	1465	-	-	-	-	-	0.1	-	0.1	0.1	0.2
37	Longipinene	1479	-	-	-	-	-	-	-	0.1	-	-
38	Citronellal	1486	-	13.7	-	-	-	-	-	-	-	-
39	Bicycloelemene	1489	1.1	0.8	-	-	-	-	-	0.1	-	-
40	Cycloisosativene	1491	-	-	-	-	-	-	-	-	-	-
41	*(E)*-β-Ocimene epoxide	1492	-	-	-	-	-	-	-	-	0.1	-
42	α-Ylangene	1493	-	-	-	-	-	-	-	-	-	0.2
43	α-Copaene	1501	-	-	-	-	-	1.1	0.1	2.1	0.1	1.5
44	Decanal	1503	0.4	-	-	-	-	-	-	-	-	-
45	β-Bourbonene	1531	-	-	-	-	-	-	-	-	0.2	tr
46	Camphor	1535	-	-	-	-	-	12.9	-	-	-	-
47	α-Gurjunene	1540	-	-	-	-	-	-	-	-	0.1	tr
48	Linalool	1548	0.2	0.2	-	-	-	0.3	-	-	0.1	0.5
49	β-Cubebene	1549	-	-	-	-	-	-	-	0.7	-	-
50	1-Nonene-3-ol	1550	0.2	-	-	-	-	-	-	-	-	-
51	Octanol	1557	-	-	-	-	-	-	-	-	-	tr
52	*trans*-α-Bergamotene	1577	-	-	-	-	-	tr	-	-	-	-
53	Isopulegol	1582	-	-	-	-	-	-	-	-	-	0.3
54	Fenchol	1592	-	-	-	-	-	-	-	-	-	0.8
55	*trans*-β-Bergamotene	1593	-	-	-	-	-	4.0	-	0.1	-	-
56	Bornyl acetate	1593	-	-	-	-	-	-	-	-	-	-
57	Decanoic acid, methyl ester	1597	-	-	-	-	0.3	-		-	-	-
58	β-Elemene	1601	0.4	0.5	-	-	-	0.1	1.2	0.8	-	0.1

59	α-Guaiene	1602	-	-	-	-	-	-	-	-	12.6	-
60	Terpinen-4-ol	1612	0.3	5.1	3.4	-	-	-	-	-	-	-
61	β-Caryophyllene	1614	-	-	-	3.5	-	6.1	-	0.8	0.4	3.5
62	Aromadendrene	1624	-	-	-	-	-	-	-	tr	-	tr
63	γ-Elemene	1650	0.4	0.5	-	-	-	-	0.4	-	0.1	0.1
64	(E)-2-Decenal	1658	0.5	-	-	-	-	-	-	-	-	-
65	Alloaromodendrene	1664	-	-	-	-	-	-	-	0.1	-	0.2
66	Citronellyl acetate	1665	-	5.2	-	-	-	-	-	-	-	-
67	*(Z)*-β-Farnesene	1670	-	-	-	0.3	-	0.1	tr	0.1	-	-
68	Muurola-4,11-diene	1673	-	-	-	-	-	-	-	-	0.1	-
69	δ-Terpineol	1681	-	-	0.7	-	-	-	-	-	-	-
70	α-Humulene	1689	0.1	0.2	1.0	0.6	-	1.6	-	0.3	0.7	0.3
71	Selina-4,11-diene	1689	-	-	-	-	-	-	-	-	1.0	-
72	*(E)*-β-Farnesene	1695	-	-	-	tr	-	0.1	-	-	-	-
73	Cryptone	1695	-	-	-	-	-	-	0.1	-	-	-
74	γ-muurolene	1704	-	-	-	-	-	-	-	0.2	0.6	1.2
75	α-Terpineol	1706	0.3	-	9.8	-	-	0.2	-	-	-	2.1
76	Borneol	1715	-	-	0.2	-	-	-	-	-	-	1.0
77	Chamigrene	1723	-	-	-	-	-	-	-	-	-	-
78	Germacrene D	1729	2.5	0.5	0.2	-	-	-	-	7.8	-	-
79	α-Zingiberene	1730	-	-	-	5.2	-	-	-	-	-	-
80	δ-Guaiene	1732	-	-	-	-	-	-	-	-	7.2	-
81	β-Bisabolone	1738	-	-	-	0.8	-	0.3	tr	0.4	-	-
82	α-Muurolene	1740	-	-	-	-	-	-	-	0.5	-	-
83	β-Selinene	1743	-	0.2	-	-	-	4.2	-	-	9.7	3.8
84	α-Selinene	1747	-	-	-	-	-	0.7	-	-	3.1	3.4
85	Geranial	1750	tr	-	-	-	-	-	-	-	-	-
86	(E,E)- α-Farnesene	1752	-	-	-	-	-	-	-	-	0.1	0.2
87	Bicyclogermacrene	1754	-	0.3	0.1	-	-	-	-	1.1	-	-
88	Geranyl acetate	1763	-	-	-	-	-	-	-	-	1.6	-
89	Citronellol	1768	-	1.8	-	-	-	-	-	-	-	0.6
90	δ-Cadinene	1773	0.2	-	-	-	-	1.4	0.1	2.9	1.0	2.9

91	γ-Cadinene	1779	-	-	-	-	-	0.1		1.1	0.5	0.8
92	β-Sesquiphellandrene	1783	-	-	-	4.8	-	0.1	0.5	0.1	-	-
93	7-epi-α-Selinene	1784	-	-	-	-	-	-	-	-	0.2	-
94	ar-Curcumene	1787	-	-	-	1.9	-	-	-	-	-	-
95	p-Methyl acetophenone	1800	-	-	-	-	-	-	0.2	-	-	-
96	Nerol	1806	0.1	-	-	-	-	-	-	-	-	-
97	α-Cadinene	1811	-	-	-	-	-	-	-	0.3	-	-
98	Hexanoic acid	1846	-	-	-	-	11.3	-	-		-	-
99	Calamenene	1854			-	-	-	0.2	-	0.1	0.2	0.7
100	Germacrene B	1856	0.3	0.3		-	-	-	0.2	-	-	-
101	*p*-Cymen-8-ol	1861	-	-	-	0.3	-	tr	-	-	-	tr
102	Carvacryl acetate	1887	-	-	-	-	-	0.1	-	-	-	-
103	α-Calacorene	1943	-	-	-	-	-	tr	-	-	0.1	0.2
104	Caryophyllene oxide	2017	-	-	-	0.1	-	0.9	-	-	0.1	1.3
105	Carotol	2042	-	-	-	-	-	-	1.9	-	-	-
106	*(E)*-Nerolidol	2051	-	-	-	0.4	-	tr	-	-	0.4	
107	Octanoic acid	2062	-	-	-	-	78.9	-	-		-	-
108	Cubenol	2081	-	-	-	-	-	0.1	-	-	0.4	1.1
109	1-epi-Cubenol	2088	-	-	-	-	-	0.2	-	-	0.6	2.2
110	Guaiol	2103	-	-	-	-	-	-	-	-	0.1	-
111	Spathulenol	2147	-	-	-	-	-	-	-	0.3	-	-
112	T-Cadinol	2193	-	-	-	-	-	-	-	0.4	0.7	1.2
113	Thymol	2196	-	-	-	-	-	0.3	-	-	-	
114	Turmerone	2206	-	-	-	31.4	-	-	-	-	-	-
115	T-Muurolol	2208	-	-	-	-	-	0.3	-	0.5	1.0	0.9
116	δ-Cadinol	2218	-	-	-	-	-	-	-	-	0.2	0.7
117	α-Muurolol	2222	-	-	-	-	-	-	-	-	0.1	0.2
118	Carvacrol	2227	-	-	-	-	-	17.9	-	-	-	-
119	2-Heptadecanone	2240	-	-	-	-	-	-	-	0.5	-	-
120	Elemicin	2242	-	-	-	-	-	-	1.7	-	-	-
121	α-Eudesmol	2246	-	-	-	-	-	-	-	-	-	0.4
122	α-Cadinol	2254	-	-	-	-	-	0.4	-	1.2	0.8	1.9

123	Guaia-6,10(14)-diene	2261	-	-	-	-	-	-	-	-	0.6	-
124	Turmerol	2270	-	-	-	14.6	-	-	-	-	-	-
125	Decanoic acid	2273	-	-	-	-	1.7	-	-	-	-	-
126	Selin-11-en-4 α-ol	2279	-	-	-	-	-	-	-	-	3.8	3.6
127	ar-Turmerone	2282	-	-	-	16.1	-	-	-	-	-	-
128	Myristicin	2291	-	-	-	-	-	0.2	40.3	0.3	0.2	-
129	Apiol	2508	-	-	-	-	-	-	1.5	-	-	-
130	9-Octadecanoic acid	3200	3.9	-	1.2	-	-	-	-	3.1	-	-
**Total identified (%)[d]**			99.5	99.6	99.6	94.2	99.3	98.0	98.6	98.1	92.6	95.8

CAL: *Citrus aurantium* leaf, CAF: *Citrus aurantium* fruit (peel), CC: *Cinnamomum camphora;* CL: *Curcuma longa,* MC: *Morinda citrifolia,*

PA: *Plectranthus amboinicus*, PC: *Petroselinum crispum*; PS: *Pittosporum senacia*; SC: *Syzygium coriaceum*; SS: *Syzygium samarangense.*

[a] Relative retention indices (RRI) calculated against *n*-alkanes (C8-C30).
[b] Not detected.
[c] Trace (<0.1%).
[d] % components identified within EOs.

References

[1] Jugreet, B.S., Mahomoodally, M.F., Sinan, K.I., Zengin, G. and Abdallah, H.H. Chemical variability, pharmacological potential, multivariate and molecular docking analyses of essential oils obtained from four medicinal plants. *Ind Crops Prod* **2020**, *150*, p.112394.

[2] Jugreet, B.S., Kouadio Ibrahime, S., Zengin, G., Abdallah, H.H. and Mahomoodally, F.M. GC/MS Profiling, In Vitro and In Silico Pharmacological Screening and Principal Component Analysis of Essential Oils from Three Exotic and Two Endemic Plants from Mauritius. *Chem Biodivers* **2021**, 18, p.e2000921.
